# From Learning Psychiatry to Becoming Psychiatrists: A Qualitative Study of Co-constructive Patient Simulation

**DOI:** 10.3389/fpsyt.2020.616239

**Published:** 2021-01-08

**Authors:** Andrés Martin, Indigo Weller, Doron Amsalem, Ayodola Adigun, Debbie Jaarsma, Robbert Duvivier, Marco Antonio de Carvalho-Filho

**Affiliations:** ^1^Child Study Center, Yale School of Medicine, New Haven, CT, United States; ^2^Standardized Patient Program, Teaching and Learning Center, Yale School of Medicine, New Haven, CT, United States; ^3^Center for Educational Development and Research in Health Sciences (CEDAR), LEARN, University Medical Center Groningen, Groningen, Netherlands; ^4^Bioethics Program, Harvard University, Cambridge, MA, United States; ^5^Tel-Aviv University Faculty of Medicine, Ramat-Aviv, Israel; ^6^Mental Health Services, Columbia University Irving Medical Center, New York, NY, United States; ^7^Parnassia Psychiatric Institute, The Hague, Netherlands; ^8^School of Medical Sciences, University of Minho, Braga, Portugal

**Keywords:** medical education, patient simulation, reflective practice, community of practice, psychiatry training

## Abstract

**Objectives:** Co-constructive patient simulation (CCPS) is a novel medical education approach that provides a participatory and emotionally supportive alternative to traditional supervision and training. CCPS can adapt iteratively and in real time to emergent vicissitudes and challenges faced by clinicians. We describe the first implementation of CCPS in psychiatry.

**Methods:** We co-developed clinical scripts together with child and adolescent psychiatry senior fellows and professional actors with experience performing as simulated patients (SPs). We conducted the simulation sessions with interviewers blind to the content of case scenarios enacted by the SPs. Each hour-long simulation was followed by an hour-long debriefing session with all participants. We recorded and transcribed case preparation, simulation interactions, and debriefing sessions, and analyzed anonymized transcripts through qualitative analysis within a constructivist framework, aided by NVivo software.

**Results:** Each of six CCPS sessions was attended by a median of 13 participants (range, 11–14). The first three sessions were conducted in person; the last three, which took place during the COVID-19 pandemic, via synchronized videoconferencing. Each of the sessions centered on clinically challenging and affectively charged situations informed by trainees' prior experiences. Through iterative thematic analysis we derived an alliterating “9R” model centered on three types of Reflection: (a) *in* action/“while doing” (Regulate, Relate, and Reason); (b) *on* action/“having done” (Realities, Restraints, and Relationships); and (c) *for* action/“will be doing” (with opportunities for Repair and Reaffirmation).

**Conclusions:** CCPS is an experiential approach that fosters autonomous, meaningful, and individually tailored learning opportunities. CCPS and the 9R model for reflective practice can be effectively applied to psychiatry and have the potential to contribute uniquely to the educational needs of its trainees and practitioners.

“As Kubie describes in his timeless paper on the retreat from patients (1), there is a difference between learning psychiatry and the process of becoming a psychiatrist. The latter is not something that a learner can gain only from books, lectures or abstract conversations, but rather from living “…within themselves the turmoil of personal growth and change, induced by repeated experiences of interacting with and adjusting to patients as they change ”” (2).

“The ability of mental health simulation to bridge the gap between education and clinical practice, alongside its potential for interprofessional education and initial evidence supporting its effectiveness, merit its inclusion as a key educational tool in providing better care for mental health needs…This integration should span undergraduate and postgraduate education, and continuing professional development across health-care settings and professions. Mental health simulation is poised to have a positive effect should the necessary support, funding, and progressive thinking be applied” (3).

The use of simulation in psychiatric education and training is a relatively new endeavor when compared to other branches of medicine. Standardized patients were first introduced into medical education by neurologist Howard Barrows in 1963 (4), with simulation in evaluation dating back to 1975, when the Objective Structured Clinical Examination (OSCE) was first described by Ronald Harden (5). Psychiatric OSCEs began to be implemented and studied systematically in the mid 1990's. This effort was led by the Psychiatric Skills Assessment Project (PSAP) group at the University of Toronto, which was later codified by Brian Hodges (6) in a milestone 2002 special issue of *Academic Psychiatry* entirely dedicated to the psychiatric OSCE.

Standardized patient simulation (SPS) in psychiatry has a number of applications in education, outside of their role in OSCEs. For example, through SPS learners can gain exposure to a wider variety of patients and clinical scenarios, and educators can reliably assess specific diagnostic or therapeutic skills (7). SPS can also provide education and assessment opportunities in graduate medical education (GME) in psychiatry, following the broader education transition from a milestone-based approach to a competency-based medical education (CBME) paradigm based on entrustable professional activities (EPAs) (8). There are a few informative examples of SPS-based psychiatric training modules in GME, including for suicide risk assessment among pediatrics residents (9), or for sexual health education specifically tailored for child and adolescent psychiatry (CAP) (10).

Despite these and other applications of SPS in psychiatry, Brenner (11) has cautioned that patient simulation may not be as effective in helping trainees refine empathetic and psychotherapeutic skills and cultivate a collaborative clinical attitude. To the contrary, we propose that the uniquely interpersonal nature of psychiatry lends itself for an especially robust application of simulation. We buttress this position through the first application to psychiatry of the recently described co-constructive patient simulation (CCPS) model (12). CCPS is “a novel approach to engage learners in a way that equally values the cultivation of their professional competencies alongside a compassionate reckoning of their challenges during medical education. The model encourages shared learning guided by the specific needs of the learners themselves, rather than the pedagogical assumptions of their instructors” (12).

A recent review (13) of simulation in undergraduate medical education (UGME) in psychiatry identified 63 studies, 48 of which included SPS. The authors applied Kolb's Learning Cycle (14) to the retrieved studies, and found that although all studies provided a concrete learning experience (Stage 1), only 19 included opportunities for reflective observation (Stage 2), 2 for abstract conceptualization (Stage 3), and just a single study for active experimentation (the final Stage 4). CCPS is experimental, experiential, and incorporates reflective practice (15) by design. As a group-based exercise, CCPS may foster the development of a community of practice (CoP) (16, 17), in which participants work together to solve a common problem (*engagement*); try to determine how an expert—or their future, more experienced selves— would solve the problem *(imagination)*; and, lastly, seek creative solutions based on shared values, which can then be enacted in accordance with the personality and style of each learner (*alignment*).

We hypothesized that CCPS can help learners develop their professional identities, both as more reflective practitioners and as a community of practice—in short, to advance their growth in *becoming* psychiatrists.

## Methods

### Co-constructive Patient Simulation

We developed and have previously described CCPS (12). In summary, CCPS is a learner-centered and experiential approach in which a designated learner (hereafter the “clinician”) creates a case script based on a challenging clinical encounter faced during training or clinical practice (18, 19). The case script is then used by a professional actor with experience working as a simulated patient (SP) in medical settings. Following established best practices (20), SPs are able to bring to life a wide array of clinical situations. A supervisor with experience in the CCPS model is involved in the creation and editing of the case script, and in the development of the simulated case. During the preparation of the script, learning goals are jointly elaborated and refined by the triad of clinician, supervisor, and actor. Case preparation includes a rehearsal, during which the SP can optimize the accuracy of their portrayal, and in which the clinician has an opportunity to re-enact and further reflect on the challenging scenario. In this context, only the clinician, the supervisor and the SP are privy to the specific details of the case. Following creation and rehearsal of the case, two fellow learners (peers or blinded supervisors, hereafter “interviewers”) with no prior knowledge, apart from a door note with brief background information of the case, will go on to interview the patient embodied by the SP. The clinical encounter is followed by a group debriefing session involving all learners: beginning with the interviewers' experiences, followed by the accounts of the clinician and peer learners, and ending with that of the de-rolled SP.

### Study Participants

We conducted a series of six simulation sessions over as many months. Participants were physicians enrolled in the final year of their ACGME-accredited fellowship program in child and adolescent psychiatry (CAP) at the Child Study Center of the Yale School of Medicine. In collaboration with the fellowship program's training director, the project was designed to provide a formative, rather than a summative educational opportunity. As such, it was intended to consolidate and refine advanced communication, diagnostic and psychotherapeutic skills gained during postgraduate training in psychiatry residency and CAP fellowship.

### Data Collection, Qualitative Analysis, and Theoretical Framework

We conducted simulation sessions with interviewers blind to the content of the case scenarios. Each hour-long simulation was followed by an hour-long debriefing session with all participants. Participants were aware that all components of each session (preparation, clinical interaction, and debriefing) were recorded and transcribed verbatim. Deidentified transcripts were then uploaded for software-supported analysis using NVivo 12 (QSR International, Melbourne, Australia).

We analyzed the transcripts using thematic analysis (21, 22), which provides theoretical freedom and flexibility to identify commonalities, and in which writing and analyzing data occur recursively alongside one another. Thematic analysis includes a rich and detailed account of the data and is considered an *inductive* approach insofar as it builds from data up to theory, rather than moving from pre-existing theory down into supporting data, as would be the case in a deductive approach. Our analyses were framed within a constructivist framework, which welcomes and encourages attention to *reflexivity* (23) or the investigators' personal and subjective views. Two authors (AM, IW) worked independently to identify and compare codes before removing redundancies, sharing them with the other investigators for further refinement, and finalizing them into a joint codebook of overarching themes until reaching *theoretical sufficiency* (24), the point at which additional data does not contribute further to the development of a given theme, or to the creation of a new one. Each key theme was supported by multiple quotes. In keeping with the tenets of participatory research (25), we value the perspective of all involved learners, and invited them to review and comment on our final codes, overarching conclusions, and manuscript draft.

We used Donald Schön's classic work on reflective practice (15) as a theoretical framework (26). This approach allowed us to consider: (A) Reflection *in* action, the metacognitive understanding of what is being done while doing it, and that allows behavioral modulation and course correction in real time; (B) Reflection *on* action, the reappraisal of performance after it took place, with a quest to understand what, how, and why it did or did not work; and (C) Reflection *for* action, the anticipation of future performance, with a deliberate search for practical, cognitive, or emotional points of improvement. We benefited for the visual depiction of our model from the application of Schön's theory to the ontology of design (27), through which the three stages of reflection respectively correspond to: the actual world (*as experienced*; “*while doing*”); to the world as understood and made sense of (as *interpreted*, “*having done*”); and the world as prepared for (as *anticipated*, “*will be doing*”).

### Ethics Approval

We obtained institutional review board approval from the Yale Human Investigations Committee (Protocol # 2000026241). Trainees were encouraged to participate but informed that their participation was neither mandatory nor relevant to their fellowship performance evaluation. They were aware that sessions would be conducted as part of a research project. All participants consented to participate in the study.

## Results

We invited all 12 graduating CAP fellows in the class of 2020 to participate, with 11 (92%) of them joining. Other participants included seven different professional actors (one for each session, except for the final one, which involved two SPs for a father-son scenario), and four supervisors. The latter included three individuals not previously known to the trainees: a physician with expertise in medical education and no formal training in psychiatry (MC), a psychiatrist with experience working with SPs (DA), and an expert in narrative medicine (IW). The fourth, a child psychiatrist and medical educator well known to the fellows as their supervisor and associate training director (AM), served as blinded interviewer in two of the six sessions. Each of the six sessions included a median of 13 participants (range, 11–14); fellows attended a median of five sessions each (range, 3–6).

### Clinical Case Scenarios

Topics that are difficult to openly talk about proved especially appropriate for the CCPS model: without overt guidance or solicitation, the scripts developed by learners in this series involved medical errors (whether actual or perceived); racial tensions, including implicit bias and overt racism; inter-professional conflict; transphobia; patient-on-provider violence; sexual health; and the sharing of vulnerability and personal imperfections in the clinical setting. We summarize the scenarios and clinical tasks for the six-case series in Table 1.

**Table 1:** Series of co-constructive patient simulations in psychiatry.

**Session**	**Case scenario**	**Clinical task**
I	Initial outpatient appointment for a young father at imminent suicide risk	Conduct a risk assessment and act accordingly
II	HIV-positive staff member files an incident report after being attacked by an adolescent patient	Determine what reporting requirements may apply
III	Hospitalized adolescent discloses physical abuse and being compelled to traffic drugs	File a report with child protective services
IV	Sentinel event and xenophobia during Inpatient hospitalization at the time of the COVID-19 pandemic	Respond to a parent's request to file a complaint
V	Difficult to engage adolescent discloses a recent rape and early pregnancy	Refer patient to appropriate clinical care at an obstetrics-gynecology clinic
VI	Sexual health discussions with a teenager, his developmentally impaired twin brother, and their father	Address sexual side effects of psychotropics; provide counseling on sexual health for a non-verbal adolescent with autism

*Sessions 1–3 were conducted in person; sessions 4–6 through synchronous videoconferencing using Zoom*.

Six clinicians each developed a detailed case script. As a representative example, the script for the second case in Table 1 is included as Supplementary Material. For illustrative purposes, that same case is deconstructed in Table 2 into the six distinct constituent phases of the CCPS model.

**Table 2:** Development of a co-constructive patient simulation session in psychiatry: an applied case example (Case II from Table 1).

**Phase**	**Applied case example**
I. Clinical encounter	• During an initial encounter, a moonlighting clinician (CL) attempts to establish an empathetic role with a staff member (SM), a bemoaning, distrusting, sporadically-coughing, grievance-seeking female, with an undisclosed HIV-positive status.
	• SM is demanding to speak to a “higher-up,” after being assaulted by a male, gender-exploring youth. She does not agree with what she considers to be an overly lenient behavioral intervention for her aggressor.
	• In an effort to establish rapport, CL listens intently, substantiating SM's feelings, encouraging her detailed, and personal experience of the encounter.
	• During the interaction, SM suggests her disapproval of the youth's gender status, referring to him as “it,” and hinting to his being “sick.”
	• Unprompted, and in a seeming non-sequitur, SM reassures CL that “there was no blood exchange during the attack.” She also brings up, indirectly and unbidden, her own past traumatic experiences, which seem to have contributed to her embittered state. SM eventually discloses her HIV-positive status.
	• The encounter leaves CL unsure about her role as a supervisor and as a clinician, even if she was “just moonlighting.”
	• CL is uncertain about her reporting responsibilities after being told of SM's HIV-positive status.
II. Reflection (*on* action)	• Long after the index encounter took place, CL remains unsettled with her overall interaction with SM. The fact that SM seemed consoled and relieved after their interaction leaves CL feeling all the more confused: “Why am I feeling worse if she is feeling better?”
	• CL reflects back to her discomfort with how SM constantly referred to the youth as “it.”
	• CL recognizes having struggled in the moment with balancing: (a) empathizing with SM; and (b) being true to her own civic duty to dismantle the observed prejudice that SM showed toward the youth.
	• CL felt “stumped” about her medico-legal responsibilities, if any, of reporting to hospital leadership about SM's HIV-positive status.
III. Script writing	• CL writes a first draft based on her recollection of the encounter, which incorporates the hybridized account of two previous clinical interactions.
	• That initial draft is shared with a standardized patient (SP) and a supervisor (S) familiar with the CCPS model.
	• Email exchanges with queries for clarification ensue.
	• Two weeks later, CL, SP, and S meet in person to further elaborate and clarify the script, as well as to role play the scenario.
	• During the role play, CL conducts the interview, and the script and the stage and acting directions are adjusted in order to maximize the emotional verisimilitude of the encounter.
IV. Simulated encounter	• Review of ground rules for the session and reading of door note (15 min).
with peer(s)	• Two peers (*P*_1_ and *P*_2_) take turns interviewing the SP-in-role (SP_IR_).
	• Each peer conducts a 20 min portion of the interview.
	• Session flow is continuous, with no break between interviewers.
	• No time-outs or rewinds occur during the interview portion of the session.
	• There is a 5 min break built into the session before debriefing starts.
V. Simulated encounter with blinded supervisor	• Phase V is a variation of phase IV, its only difference being the participation of a supervisor blind to the case scenario (S_BL_) as one of the interviewers on the “hot seat.”
	• In our CCPS series, four sessions were conducted with peer interviewers only (IV), and two with an S_BL_ (V) serving as one of the interviewers.
	• For this particular session, only P_1_ and P_2_ served as interviewers (i.e., there was only a phase IV, but no phase V).
VI. Debriefing	• P_1_ and P_2_ begin by sharing their experience, including how they felt while being on the “hot seat” and under the watch of their peers and supervisor(s).
	• Other peers (P_3_-P_n_) and supervisor(s) share their observations and personal reflections.
	• The facilitating supervisor (S) aims to have most of the debriefing content come from P_1_-P_n_ but provides input and guidance to steer the discussion, following best medical education practices in debriefing.
	• The scriptwriter (CL) shares only near the end of the debriefing and is encouraged to share just how realistic the scenario was, and what reflections were elicited in seeing a peer (or a supervisor) deal with the “same” patient in the simulated scenario.
	• SP, silent up to this point, is introduced in her re-rolled self, and goes on to share her experience and reflections while being in the role of SM.
	• For this particular scenario, some of the salient themes included: ° Challenges to establish empathy and ascertain trustworthiness with an off-putting, help-rejecting, and angry SM.
	° Balancing attentive listening and asking of salient questions with a hyperverbal, demanding, and irritable SM.
	° Welcoming/being aware of SM's emotional reaction regarding her aggressor, while also being aware of, and not allowing one's own countertransference to take over.
	° Establishing effective communication dynamics and build rapport despite one's own charged response and anger to SM.
	° Serving in multiple roles at once, including as a supervisor trying to resolve conflict between SM, other unit staff, and youth patient.
	° Communicating confidently and accurately regarding SM's HIV-positive status vis-a-vis the hospital's administration.
	° CL acknowledges uncertainly about her medico-legal reporting responsibilities, if any, after being informed that the staff member has HIV. She learns in the debriefing that her colleagues were similarly unsure.
	° Articulating where such information could be obtained (e.g., through a confidential inquiry to the legal or human resources office) and feeling comfortable with not having all answers readily on hand (e.g., “I don't know what may be my responsibility here, but I will inquire and consider possible next steps, if any.”)
	° Performing a risk assessment to ensure SM's wellbeing.
	° Validating the emotions and mental/physical state of SM, while also acknowledging the emotions and mental state of the youth.
	° Being flexible with adjusting previous behavioral intervention plans for a youth, after listening to SM's account.
	° Reassuring SM that her communication with the clinician is confidential and will not affect her job status.

### The 9-R Model

**Figure 1:**
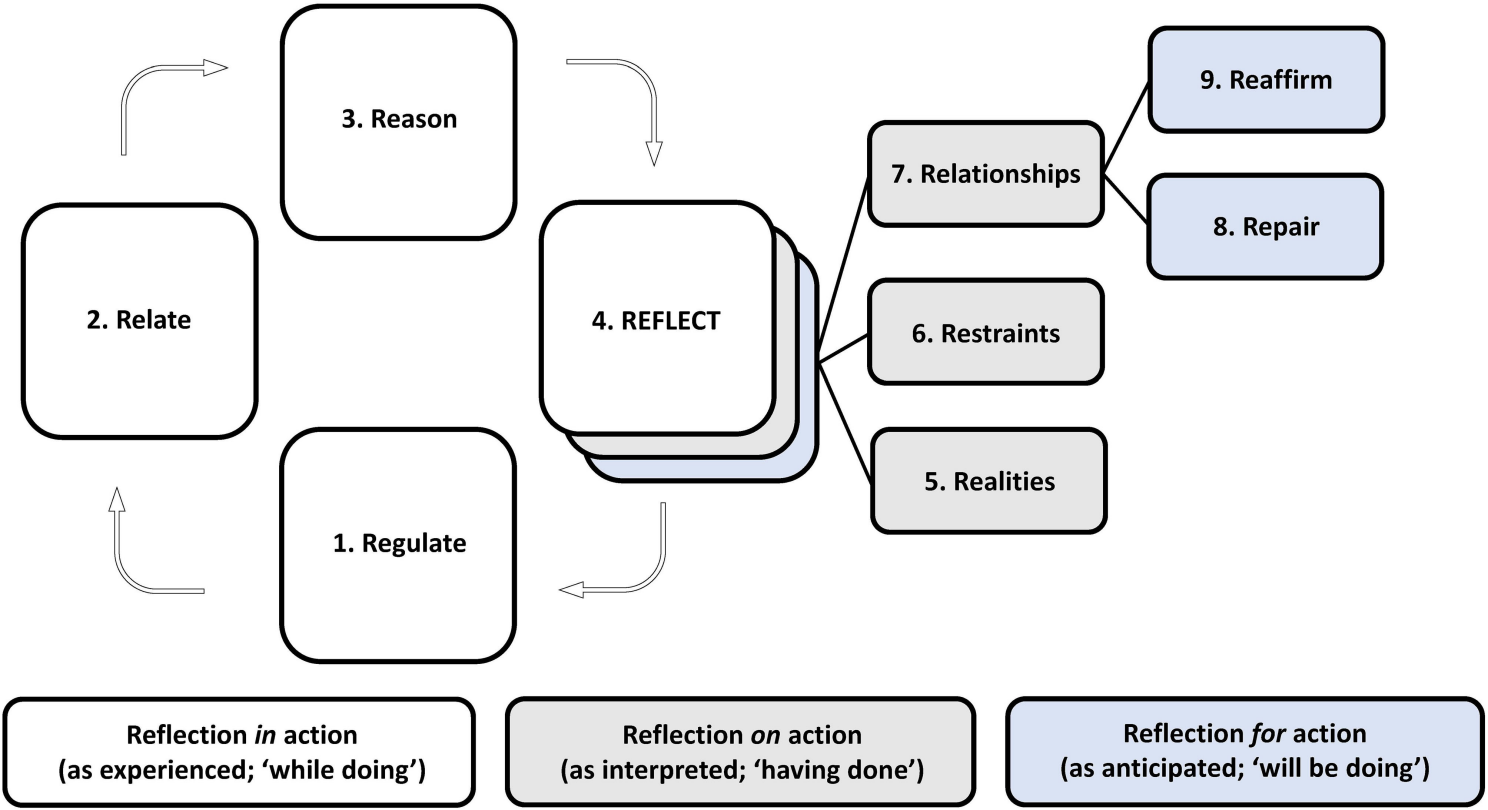
The ‘9R' model for reflective practice.

Through iterative thematic analysis, we developed an alliterating “9R” model depicted in Figure 1, the components of which we go on to describe in turn. Reflection is placed at the center of the model, as it is by reflecting on the simulation session and connecting it with previous and likely future experiences that the entire CCPS exercise is set in motion.

**A. Reflection *in* action: the world as experienced (‘while doing')**

The left half of Figure 1 has three distinct phases flowing out of Reflection and streaming back into it: (1) Regulate; (2) Relate; and (3) Reason—terms that we adapted from Bruce Perry's neurosequential model of therapeutics (28).

Supporting quotations in this section and the two that follow are attributed using the convention: *Session* (I–VI), followed by *Participant* (1, 2, 3, *n*); the letter “S” is appended at the end when the speaker is a supervisor; the letters “SP” when a simulated patient.

1. Regulate: don't go limbic

We encouraged clinicians to think back to emotionally charged and challenging patient interactions when considering experiences to base their scripts on. Clinicians crafted cases in which actors expressed emotions that included, among many others: anger, contempt, disgust, resentment, fear, pity, reluctance, sadness, suspicion, and raw emotional intensity. Interviewing clinicians had a dual task before them: to both welcome and muffle emotional extremes in their patients as much as in themselves in order to proceed with the specific task at hand. Two of the interviewers described the initial moments of the encounter as ethereal and almost dreamlike:

I think I was trying to moderate her [the SP's] high tone and missing out some details in the process. It was all very volatile, like going up, and down trying to contain her affect. The feelings were really long lasting: the mix of confusion, of containing her aggression, anger, and shame. And it felt at the debriefing session like I was still living in a fog. I couldn't really process it all, not until now (II.4).

At some point, the tension was so palpable that I felt the background drop off, and it had become just him and me. It was too hot, back off: come back to it when the affect is down (III.1).

The source of the intensity could be free-floating and hard to pin down to a precise source, although at times there were clearly recognized triggers:

I really struggled with what my own feelings were about, but when she called the patient “it,” I had really strong feelings. I had no idea how to. When that went down, and it's all happening I just was not.on a human empathy level, I was not connecting with her. Because of this, it was hard to sit through. Being genuine with myself I just didn't know how much longer I could go listening to her (II.1).

The emotional charge in the room was strong enough to overflow from the interviewer-patient pair to those witnessing the interaction from the sidelines. As one of the witnessing peers recounted during the debriefing:

You guys saw me with my hair down. It was just too much emotional input. I had to kind of go down and try to limit my vision and other sensory input to just calm down because it was all so difficult to hear. It was just so hard to sort out the systems part from the patient part because my emotional response was so overwhelming (II.5).

Efforts to defuse or de-escalate a patient's raw emotion were variably successful, with interviewers recognizing that certain ministrations may have been directed to themselves in a failed effort to gain control:

I used the words, “That was really frustrating” a couple of times. I should have waited until I got more. “Frustrating” is a word we love because it makes us feel less uncomfortable. We're afraid of patients being furious. It's what you say to a toddler who is rageful, “Oh, are you frustrated?” That's what they teach you. “Are you afraid?” “No, mom: I want to murder you. I hate you mommy, I hate you.” (II.2)

Sitting with the SP in quiet presence, listening attentively, and allowing silence into the interaction was often more effective. The newfound awareness to resist the urge to provide stock commands and empathic responses to the patient when under pressure was also revelatory. Here, silence could be construed as an active and intentional invitation, rather than the mere absence of speech:

To hear before rushing in to validate: that part does not come so naturally to me. I have to remind myself—“Don't just *do* something: *sit* there” (IV.2).

She opened up the most when you remained quiet. You gave her a big chunk of space, when it was all quiet. And that's when she started opening up about her HIV status. Because emotions can rise so high, we sometimes want to stop them, or to stop her, and to try to interject with “I hear you,” or “just sit down,” or to say anything at all. And sometimes the best thing is to just stay put and do nothing. It sure seemed to take effort (II.3).

No matter what type of words we use for empathy, to see her [fellow peer's] body language as an interviewer, and to sense her presence itself, was much more powerful than the use of any words. Seeing her do it, seeing her just being present and actively listening was remarkable (IV.4).

Simulated patients responded to such a “less is more” approach:

As a clinician, you were disarming to me as a parent. I wanted to be angry. In my script, I was ready to yell and scream, to swear. But you seemed to be really listening to me, and to care, so I could not muster any of that. It would not have come out as legitimate; it would have been a cartoon (IV.2SP).

2. Relate: it is difficult to be angry at someone who treats you with respect

It was only once the affective charge in the consultation room was dialed down that the interviewer could engage the patient's plight with an attitude of compassionate curiosity. Depending on personal style, areas of affinity or commonality, or on a pre-existing clinical relationship, clinicians were able to express empathy, humility, humor, vulnerability, or understanding, all in the service of connecting in such a way that could be received and returned in kind.

I couldn't imagine the guilt, the shame or the things that she must've been holding and feeling, and the way she must have run to the bathroom to check and make sure there was no blood to see, “Okay, did I transfer it [HIV] to him?” And that should not be something that she should carry alone. But that's what she has been talking about, not having support from the workplace (II.7).

The unique circumstances of the COVID-19 pandemic organically led to finding common ground:

As a therapist, all of a sudden you are on the same pandemic boat in that they are also locked in and sheltered in place. And so we are all undergoing a common stressor, and that is unusual in our work as therapists (IV.2).

Although certainly unusual outside of a shared disaster situation, the prolonged uncertainty of the pandemic crisis facilitated a leveling of the emotional playing field that can undergird meaningful human connection.

To feel the commonality, rather than just talk about how that is impacting them and not considering your own experience. This is really hard but necessary: we're all going through a lot of stress (IV.3).

3. Reason: keep it frontal

Each of the six case scenarios was yoked to clinical tasks to be completed by the interviewers/fellow learners that the writers/clinicians felt they hadn't been able to complete when they had faced similar challenges in the past. For the educational purposes of this CCPS series, such task-based goals were of secondary, though not negligible importance.

The need to balance maintaining connection (which spans regulating and relating) with performing certain tasks or deliver specific content (reasoning) is not unique to the setting of an “artificial” simulation session. Indeed, it is inherent to all clinical practice, and as such, an important one to revisit and recalibrate periodically. One learner

…worried about guns, now that you mention it. I considered asking, I wanted to push more. But at the same time, I see now that I was afraid to make him shut down. This was a hard situation. And I think that the shifting of gears from, “I'm in outpatient therapist/“empath” mode” to “I'm in emergency room/“do stuff” mode” proved to be a challenging switcheroo (I.8).

That interaction was experienced in a contrasting way by the de-rolled actor, as he described after the debriefing session:

SP: The interviewers seemed so tentative and scared. And yet they weren't paralyzed. That is what blew my mind.

S: Interesting choice of words, right?

SP: They were so scared, yet no one asked if I had a weapon. Did they think that because it is an outpatient private practice setting no one could think of using a weapon? They just seemed so concerned about my coming back and my wife coming back. I felt like saying, “Hey my wife is going to come back as a widow.”

   (I.8SP, I.9S)

As sessions progressed, clinicians were better able to navigate these competing needs, and to more seamlessly progress across the first three steps. Greater familiarity with the CCPS model was certainly one reason. Another was the collective ability to become more relaxed while “performing”—not just in front of each other, but in softening the edges of their idealized clinical selves, which loomed large as their graduation date fast approached.

I realize now that I had been so focused on connecting with her. Clearly at the expense of carrying the ball forward. For a moment there in the middle I did carry it forward a bit, before getting stuck again (V.9).

As learners eased into the exercise, they gained intentionality in their actions, a future-forward reflexivity akin to scenic intelligence:

It was like working along two axes at once. There was the “connecting” axis, you know, how much or how little, minute by minute, or during an hour, how much you are connecting at a human level. And a whole other axis of “getting business done.” They both needed care and feeding (V.3S).

Whether we ultimately got the answers “right” or “wrong,” no one will really care. That part is less relevant, more forgettable, and more traditionally “teachable.” It's less about that, and more about how this experience will inform us in the future. And I for one know that I've learned a lot just by being at these sessions (VI.1).

**B. Reflection *on* action: the world as interpreted (‘having done')**

4. Reflect: pressure—how diamonds are made

Reflection is the central node of the 9R model, and one that is iteratively deepened throughout the cycle. As such, it is not entirely possible to disentangle it from the other components or timescales. This is exemplified in the longer quote that follows (I.10S), in which the simulation session, for the supervisor-turned-scriptwriter, serves as a point of departure for a reckoning over past clinical years. It starts with a reflection in the present, as the encounter is witnessed in real time, followed by what blind spots it revealed as the memories intimately bound to the case were “brought back to life:”

I was so intensely rooting for them in my mind. I wanted them to get it right. We all knew that this wasn't “for real,” that this was not being scored or evaluated for any kind of high stakes evaluation. And yet, it was *personally* important for me that they succeed. So I was really taken aback and saddened when they didn't, because it felt, and this is may be the key point, it felt like it was *me* who was failing.

From that key moment of intersubjectivity, the scene hearkens back to the past and its interpretation; to what led to the initial writing of the script in an effort to recontextualize earlier failures, whether real or perceived:

This is what made the connection become visceral, because it was only after the case was complete and after seeing them “fail” in this part of the exercise that I realized that on not one but on two occasions in my professional career I had failed exactly in the same way that they had. And this realization had been lying dormant and forgotten somewhere in the back of my mind. Until then. Until I saw them in action. This was maybe a good 20+ years ago and I hadn't thought of it until seeing them squirm. They squirmed in the very same way that I had squirmed so many years before.

Anticipation informs future action, as the experience is metabolized and put to use toward new clinical and teaching opportunities:

I only touched on the surface when telling them that “I have been where you were today,” but I didn't fully share, not really. Not just because time ran out, but because I wasn't really aware then of what had just happened. I am committed to doing better in the future. And I certainly hope that in sharing this now and in the future, I will be able to close the loop.

Soon after emerging from the discomfort of being on the “hot seat,” an interviewer captured the reflective essence of the simulation:

We are learning. These exchanges are our teacher. I am grateful. I am open. I am hopeful that talking about vulnerabilities and hardships can apply pressure for us to think deeper, to delve deeper. Pressure. That's how diamonds are made after all (II.8).

The active gerund “learning” and the emphasis on the collective in the interpretation of the recent events mark a shared and ongoing process. Equally, the invocation of the geologic timescale of a gemstone serves a reminder of the enduring struggles of professional development; one in which the heat and pressures of what was once left unsaid is precisely what makes the experience a precious resource for the group.

5. Realities: viruses do not discriminate, but society does (29)

Even prior to the pandemic, participants had already confronted painful realities not only through the development of their own scripts, but by witnessing their peers' and their own reactions. For example, debriefing sessions fostered discussion that moved away from merely acknowledging implicit bias and unconscious behavior to talking about overt and systemic racism in a direct way:

Being a Black doctor, I've had a lot of interactions with staff members who weren't that familiar with me, who questioned who I am, what I am. So, it's just that act of *doctoring while Black*: how do I communicate without being demeaning? I knew being Black while doctoring was real, but I never expected mirrors of my skin to invite more scrutiny than my actual self. Realizing that my physiology beams during these instances due to fear of rejection, to a need to be right, a need to be trusted, a need to be a beacon to my people—the minority. I am praying that being a doctor is no longer a surprise (II.14).

The patient depictions and ensuing interactions were evocative enough to prove stressful and morally confronting in the moment. But the exercise and its debriefing also afforded participants enough psychological distance to permit self-examination in a nurturing setting with sufficient time and supportive guidance. This made it possible to confront and discuss painful realities in a less activated and more considered manner than under the automatic fight, flight, or freeze responses that are so often ignited at these moments.

“You people.” I think I wanted to hear, “you *doctor* people” from her. But then she mentioned the chip on her shoulder, so I thought maybe she meant “you *educated* people.” But that was just my not wanting to hear it, my wishful thinking. It was “you *Black* people” who she meant needed to go somewhere else. We needed to go to a separate unit—to a “Black unit” (II.12).

One of the strengths of the CCPS model is its ability to adapt iteratively and in real time to daily clinical vicissitudes. This became palpable during this six-part series, bisected as it was by the onset of the COVID-19 pandemic. As a case in point, the fourth session was initially designed around actual events experienced by the scriptwriter, who had confronted a parent seeking legal redress after the clinical team failed to ensure the safety of her adolescent daughter Emma, resulting in a serious suicide attempt while hospitalized as an inpatient. A few days before the simulation took place, the setting was adapted to the new realities:

Scenario takes place while you are moonlighting as the on-call psychiatrist at an inpatient adolescent unit. Your institution has adopted the CDC guidelines during the COVID-19 pandemic: practicing social distancing, minimizing clinical staff on the unit, and communicating with patients and families through virtual online and telepsychiatry systems. You are about to get a Zoom call from Emma's very upset mother (From door note, session IV).

In very short order, we had to not only move the site of delivery from a brick-and-mortar location to a virtual one, but to address the many challenges posed by the viral crisis. Events and guidelines early in the pandemic changed on a daily basis and informed the content of the session, by which time the popularized claim of the virus's origin had reactivated an array of racist and xenophobic tropes with profound implications for Asian–Americans. Critical discussions around xenophobia and racism, along with the pandemic's grave elucidation of pre-existing health inequities became not just inevitable, but of urgent necessity.

Emma is a bi-racial and bi-cultural (Chinese–American) 14-year-old adolescent girl who presented with depression and a serious suicide attempt following a racial cyberattack by her classmates: they had circulated an offensive meme of a “Chinaman” eating soup overflowing with bats and pangolins, and said that Emma surely ate the same “Chinese porridge” at home. They called her a “mongrel” and said that she, her father, and “their like” were responsible for spreading the “dirty, nasty China virus” (From door note, session IV).

6. Restraints: life happens

Over the course of the series, a number of imperfections, factual errors, anachronisms, or verbal missteps inevitably took place. Rather than dismissing these instances as events that diminished the verisimilitude of the clinical scene, we saw in them, instead, vital conduits toward growth and reflection. Disruptions in the conceptual “fourth wall” of the “stage” separating a session's “performers” from its “audience” members provided valuable opportunities, as when a participant's snoring wrenched everyone's attention away from the actor. Bashful and self-conscious as he recomposed himself, he later went on to share during the debriefing:

I'm embarrassed and so sorry that I snored, but I got no sleep: my friend died last night (III.8).

That expression of candid vulnerability and grief resonated with the group and, in turn, gave one participant permission to name their own human fragility:

You were an influence. I'm actually growing my beard because my mother died 3 weeks ago. In the Jewish tradition, we grow out our beard as a sign of mourning. So when you first came in and said, “Hey, you look great with a beard,” I thought to myself “No I don't. That's not the point.” I only mention this because you too had a loss yesterday. I had a loss recently. And as I was coming into today's session, I was thinking that I have been back to work, that life goes on. Life went on for you as well. As it did a few weeks back when one of you showed up the same day your baby had been taken to the emergency room just a few hours before. These things happen. Life happens. And how do we each compartmentalize, ignore, embrace, or move on? So I salute and thank you for snoring, as it actually reminded me that this is all real, and that half of life is just showing up. Sometimes you're exhausted, and tired, and things happen, as they have today. And yet, you are here, as you have been for your patients (III.4S).

The fourth wall could also be breached through the overwhelming emotional response to a patient interaction. At such times, affective overflow spilled over onto the group trying to contain and make sense of it. This resulted in the intersubjective blending over who exactly was taking care of whom, as exemplified by a supervisor's recollection:

You were sitting next to me and I was actually worried that you were having something medical going on. I didn't want to break the spell and say, “Are you having chest pain?” But I was really worried and hearing about the role confusion in the case made it additionally palpable: just who exactly is taking care of whom? Am I taking care of you, or being present to see how your colleagues take care of the SP? I'm so glad you were not having a heart attack, by the way. When I asked, I was relieved when you said, “No. It was overwhelming. I just needed to put on my screen” (II.7S).

In short, we consider whatever degree of “disruption” caused by the actions and experiences on both sides of the “stage” as equally important to the overall efficacy of the simulation sessions.

7. Relationships: moving from me to we

As a dyadic interaction between a clinician and an SP, CCPS is designed to provide a controlled environment in which to closely experience and examine human relationships as they are shaped and pressured by the clinical encounter. With the additional participation of fellow peers and supervisors, CCPS provides a rich setting through which to delve into what happens not only in the experiential interaction itself, but in that interaction as witnessed and interpreted by others [“moving from me to we,” in the words of a participant who may have been paraphrasing Mohammed Ali's *Me We* poem, or Dan Siegel's *Mwe* construct (30)]. We go on to describe some of the relationships that can be fruitfully explored in this simulation setting.

*Peer-on-patient*. Participants articulated and shared challenges in empathizing with patients:

My initial reaction was “This jerk. She hates trans-kids and there was nothing this kid could have done to make her feel differently.” We know she's struggling with the fact that these kids exist, but her awful language was hard to take, like calling the kid “it.” But then as both [interviewers] were expressing curiosity and not shaming her, it actually became clear that it wasn't really a trans issue. It was rather an “I'm overwhelmed, and this is a change that I'll eventually get, but it is just one too many things right now” (II.10).

The participant's overwhelmed response speaks to a broader challenge for the group in learning to manage competing roles and responsibilities that seem at odds with each other:

It became really hard to figure out what our exact role was, to remember “OK, this is a staff member, and you have some responsibility to the staff member. But there's also the patient, and you have a responsibility to the patient as well” (II.7).

*Peer-on-peer*. One of the more frequent comment types, and one articulated after virtually every session, was that of gratitude at the opportunity to see each other “in action” and to witness each other's distinct therapeutic styles:

I'm never in a room where I see my peers interviewing like this. It's rare that I see a full interview or session and just sit there and watch, being so present in the moment. I learned so much from every single session I attended (VI.5).

What really stands out to me now was how incredible it was to have the opportunity to see my co-fellows in action. I think I learned as much from watching them do their thing. Comparing their styles after having gone myself; watching them struggle with some of the same issues and challenges. And seeing them approach things from similar and at the same time unique angles was really useful, really educational (VI.6).

*Peer-on-supervisor*. The opportunity to work alongside a senior colleague and navigate the very same clinical challenge in real time was educationally potent for many:

It was so useful to see how a senior child and adolescent psychiatrist got the “job” done, and how much growth I can look forward to throughout my career. I also think the discussion made me realize that no matter what, we are going to be making mistakes after we get out of fellowship, and that there's always an ongoing need for training and continuous feedback. This felt like a supportive place to really talk about that (V.1).

In addition to seeing our peers, it was also wonderful to have the opportunity to observe faculty. It would have been a big loss to go through training and not see the wealth of experience that our faculty members have interviewing and struggling with their own patients (V.3).

*Supervisor-on-peer*. In turn, supervisors found grounding and comfort in sharing their own limitations and being able to see themselves in their trainees:

Although they may have “failed,” they failed in the very same way and at the same professional career stage that I had. I am humbled by the experience and so grateful that neither my patients nor this simulated patient lost their lives. My hope is that, if nothing else, sharing my own failure, may soften the blow of their own. There is comfort in knowing we are all similarly flawed. More importantly, that we are all similarly educatable and remediable, despite how unforgiving we may be with ourselves (I.8S).

**C. Reflection *for* action: the world as anticipated (“will be doing”)**

One of the more challenging aspects of the simulation sessions, and one that required active facilitation, was in helping participants move from only critiquing their own or each other's performance during the patient interaction toward a place of growth and trusting anticipation—to incorporate lessons from the current experience as a template for future action:

The ability of being able to see what you're doing while you're doing it is hard. There was a lot of talk about “presence,” as there should. But they needed our help in moving the session to talk about the future. Having gone through this hard experience together, how do they now think these experiences can inform their future practice? (IV.9S).

To that end, anticipatory reflection provided the two distinct sets of educational opportunities outlined next.

8. Repair: Try again. Fail again. Fail better (31)

Scriptwriters were in the unique vantage point of seeing how their colleagues or supervisors would deal with a situation that had previously challenged or outright stumped them. In this way, they were able to transform a series of difficult encounters passively experienced as failure into a single “do-over” opportunity in the service of a humbled excellence:

We are still learning and growing. The very things that make us uncomfortable now provide seeds for growth in the future. How do we choose to water them? As psychiatrists we hone into the role of problem-solver, healer, know-it-all. But what if we don't know? Is it OK to say we don't know? Will our patients forgive us? Will we forgive ourselves? (II.7).

As trainees fast approached their graduation and launch into independent practice after years of fellowship training, the yearning for competence had a particularly strong resonance, one that partly explained how

…the challenge and even discomfort of the experience was how it rubs against the idealized notion of who you are and who you want to be moving forward (VI.2S).

For peers who in turn struggled while on the “hot seat” the obstacle became their path,

…the take-home message was not that I “failed,” which I didn't, because that wasn't the point here. We each “passed” by feeling the despair, the difficulty, and how to mobilize and re-mobilize when something inside us gets in the way. I think that is where we were all at. And I see that as a big success. I know I will apply these lessons as I move forward in my practice. And to see just what an “unleashed” simulated patient can do: that part was truly remarkable (VI.3).

There were further opportunities for reflection even after the simulation and debriefing sessions were over. The enduring “afterlife” of some sessions along with the trust that had developed was exemplified in a moment of repair that followed a misnaming incident:

As people were leaving the room, I looked the way of one of my colleagues and mistakenly called her by the wrong name. My two colleagues are as different as two people can be. But they are both Black and have distinctly African names. I've never considered myself beyond reproach and am aware that we all have biases at play. But it was quite remarkable to see this take hold immediately after and in the context of our discussion on racism and bias. To be schooled so on the spot was to be served a big slice of humble pie. As if all of that wasn't enough, I checked my phone once during the break, wanting to make sure I hadn't missed anything clinically important over the past hour. And as I checked, I saw a news alert. The Administration had just extended a ban on immigrants from several African nations. I was embarrassed. Of my country. Of myself. I recognized this exercise had not been hypothetical. It was as real as real gets. I was humbled and schooled. I have so much to learn. We all do. By sharing it now I not only apologize (which I do once again here, in a heartfelt way), but see how much growth we each have ahead of us, and what a privilege it is to learn along as caring colleagues as she—and as magnanimous in her forgiveness (IV.6S).

In the session that followed, the same two individuals had to collaborate alongside each other on the “hot seat.” Having an opportunity to extend these sentiments outside of the session and formally apologize was a powerful demonstration of the protean humility and action that the model can foster:

Thank you very much for your email. It has made my day. I was almost going to make a comment then and there, but I decided it was not going to be helpful at the time, and that I could also reflect on the things elicited by being called a different name in that moment. For you to write about this and to share this openly takes an admirable level of courage and I'm inspired by it. Thank you (IV.7).

9. Reaffirm: becoming child psychiatrists, together

Each simulation session offered an opportunity for putting into practice an existing and hard-earned professional skillset. But well beyond that, each session also offered the reaffirmation of mastery developed over the course of the six-part series, as

…a safe place to revisit outdated clinical practices. When will we have time to really sit down together and process these challenging cases, not only to talk about them, but to see them unfolding in real time? I think that part was really valuable—the doing and not just the talking (VI.2).

A shared sense of recommitment became evident on at least three different planes:

*As participants*. Clinicians didn't only struggle; they also prevailed when facing the very challenges they had trained to resolve:

The stress I felt was not because I thought, “Oh my God, I'm being watched,” but rather because this was hard, truly hard. The patient interaction, I mean. And coming out “whole” on the other side, realizing I had done the very things I have spent so long training for—that gave me a very special and reassuring feeling (IV.3).

*As supervisors*. The demonstration of clinical competence by senior colleagues may have initially felt boastful, as when a supervisor shared ambivalence over

…how “well” it went for me. I don't know what, if any, my “Open Sesame” was, but even as it was happening, and even as I sensed “I've got this,” that was not what I wanted to model. I didn't want to give the illusion that I have some kind of magic. And I certainly didn't want for it to be all seamless and showy while you squirmed and struggled: I would have liked for us to share in a similar failure, to realize that we all can fail at times—as we surely do (3.8S).

By the end of the series, learners had expressed the value they found in seeing difficult situations handled by a supervisor, who in turn felt less compelled to apologize:

That time, when I had been all guilt-ridden about not having failed and, you know, wanting to fail right there along with you. I found it liberating when you said, “Oh, it's actually very helpful to see you doing it so well, because otherwise you're like way more senior, and if you don't do a better job than me, then we're all screwed because we cannot ever learn and grow.” I found that liberating: thank you (6.8S).

*As a community*. Ultimately, the series provided a shared experience for growth—not just of individuals developing and maturing as professionals, but of a group larger than its constituent parts, for

In almost every session we talked not only about this or that person's performance: we were becoming child psychiatrists, together (6.2S).

## Discussion

We were able to apply the co-constructive patient simulation approach to a psychiatric clinical context. Moreover, we arrived at a structured nine-part model for reflective practice that offers a novel way to learn, teach, and experience psychiatry. To situate this model further, we first turn to examine the role of CCPS as a means of teaching and learning psychiatry, and the distinct ways the model garners its pedagogical utility for fostering compassionate curiosity in the group. We next consider CCPS's role as a means to build and strengthen a social community of learners. Lastly, we address the model's limitations and future opportunities.

### Learning Psychiatry

In considering the potentially unique contributions and added value of CCPS, and particularly when contrasting it to traditional supervision, we apply the five “good teaching” perspectives proposed by Pratt et al. (32) as a conceptual frame through which to revisit the assumptions and beliefs that as medical educators we hold regarding learning, knowledge, and teaching. The first three of these perspectives are effectively incorporated by supervision and CCPS alike: *transmission*, or the transfer of knowledge in a way that goes beyond the mere mastery of techniques; *developmental*, or the constructivist attunement to the individual learner's unique needs and point of view; and *nurturing*, which recognizes the interaction between knowing and feeling in the learner, and that addresses vulnerabilities inherent to training, such as the fear of failure. The fourth perspective, *social reform*, refers to the fostering of ideals and values, particularly as they pertain to inequities in care. CCPS provided a setting to center issues such as racism, xenophobia, and transphobia to become readily palpable and contributed to the mobilization of its learners to address these issues in a more systematic way in their training and practice (33).

CCPS can be conceptualized as a variation of, and a synergistic complement to traditional supervision, which constitutes one of the educational bedrocks for teaching and learning psychiatry. Clinical supervision of trainees is associated with improved patient- and education-related outcomes. In turn, inadequate supervision has been repeatedly identified as one of the most common causes of medical errors. Since the early 1990s, medical specialties have required the physical presence of an attending physician during the delivery of key aspects of care by a trainee (34).

CCPS and the 9R model could provide a rubric to support the academic and professional development of individual trainees and their personal milestones, even if that was not the primary goal of this study. We believe that CCPS could stand to make a unique contribution to psychiatric education as the field moves into competency-based education (CBD) (35). CBD requires maturation beyond the current focus on education as a pass/fail model toward a developmental paradigm unique to each learner. It requires medical education to revisit its focus on the stigma of failure during the training years, and aims to create opportunities for trainees, graduates, and lifelong learners to “try again, fail again, and fail better,” as Samuel Becket would put it (31). For our field to simply adapt traditional psychiatric education methods could be a missed opportunity, as the major curriculum renewal embodied in CBD should not be a once-in-a-lifetime opportunity.

In the case of psychiatry, the “how” of supervision, largely unchanged for several decades, has been based on the teaching triad of modeling, rehearsal, and feedback (36). Supervision's “where” and “when” have been more variable, leading to four main types: (37) *case discussion*, in which the supervisor is removed in both place and time from the clinical action, and as such not privy to what actually takes place in a given interaction; *co-therapy*, in which supervisor and trainee join together in a session, to the detriment of the latter's independent action; *with delayed feedback*, in which the supervisor can, through a one-way mirror or recording technology, get a more objective sense of what took place and provide feedback at a later time; and *live*, in which the supervisor can provide input from a distance but in real time, as through a “bug” in the trainee's ear. Each of these four types can be complemented by the presence of fellow learners or supervisors, leading to *peer* or *group* variations of supervision. CCPS is in fact one type of group supervision, and one that through the sharing of space, time, and clinical interactions, results in the immediacy of reflection *in* action, *on* action, and in the mobilizing *for* action.

The “what” that is embedded in supervision can include a wide range of content. In the case of psychotherapy, for example, psychodynamic approaches have been common, if highly variable; cognitive-behavioral therapy training in psychiatry has advanced significantly over the past two decades; and even though supportive and eclectic psychotherapy is the most widely practiced, it still tends to receive the least systematic attention (38). Over the past decade, mentalization-based therapy has become increasingly popular in practice and training. Conceptually related to empathy, mentalization (or reflective functioning) is defined as “the capacity to understand and interpret—implicitly and explicitly—one's own and others' behavior as an expression of mental states such as feelings, thoughts, fantasies, beliefs and desires” (39, 40).

Through its close attention to the mental states of others (including of the professional actors in and out of their assigned SP roles), CCPS is particularly well positioned to incorporate a mentalization-based approach. The 9R model in turn provides a systematic and orderly roadmap through which to disentangle the components that inform overarching constructs as complex as mentalization. For instance, the sequence through which each participant shares their experience reveals the complex ways an event is mentalized as it reverberates within a group: from the scriptwriter who held the encounter with the original patient, through the interviewers' and actor's experience of the case, to its reception by the wider group of peers. Through the careful delineation of each perspective, participants describe the varied angles of arrival to the event so as to open clinical possibilities and bring curiosity to each other and to the clinical challenge. In one case in particular, debriefing the SP's use of transphobic language created a generative divide in the group where for one side, the language foreclosed any attempt to mentalize the patient's experience, whereas for the other side it was the SP's confusion over the appropriate language that catalyzed their mentalization. By being candid and vulnerable in giving voice to both sides while naming each constraint, CCPS grounds the theory of mentalization, and prepares trainees for the daily reality of the ways in which challenging cases often force the clinician to confront the gap between their idealized and empathic clinical self and the self that acted the best they could at the time and under pressure.

### Becoming Psychiatrists

Pratt's fifth perspective, *apprenticeship*, may evoke an over-simplified “see one, do one, teach one” prescription for acquiring skills through a relationship between apprentice and mentor (41). More fitting is the notion that teaching and learning are “rooted in the doing of work, not just [the] talking about it” (32). Psychiatric encounters are often fraught with ambiguity and uncertainty, which can benefit from the type of experiential and situated learning that patient simulation makes possible (42). We consider that CCPS provides an opportunity for such apprenticeship in a way that traditional supervision cannot. It does so through practicing the belief that teaching and learning are best done while interacting and participating in shared work; by providing a context in which knowledge is tested, questioned, and applied; and by sharing in the responsibility of creating authentic and relevant tasks, problems, and assignments in which to put one's craft, knowledge, or skills into action.

By virtue of being a group-based experience in which trainees and supervisors see each other in action—and hear each other in reflection—CCPS relies on all participants involved, and not only on those in the more active roles of clinician, interviewer, or SP. Except for the actors, who joined only one session each, participants were involved in the complete series of six simulations. This thread of continuity afforded the group an opportunity to develop a commonality of experience, for trainees and supervisors to collaborate alongside each other on the humbling task of learning and growing together, to engage in thoughtful critique of the model, and to co-create a more nuanced, shared language and approach. In this way, the “dosing” effect of a series of simulations (as opposed to a single session) needs to be taken into account: the effects over time may be more than simply additive. For example, the broad range of challenges faced, coupled with the emotional responses and individual clinical approaches used to address them, gained momentum over time and had a virtuous learning impact on all learners.

By providing shared understanding and practice, by creating a bond among the learners and supervisors, and by explicitly addressing role modeling (43, 44), mentorship, reflection, narrative humility (45), and experiential learning, a beneficial “side effect” of the CCPS serial approach was in supporting the development of a community of practice (CoP) (46). Like other successful CoPs, CCPS is a collaborative effort that seeks the co-creation of practical solutions to common problems encountered in clinical practice (47). Participants were activelly developing a CoP: becoming psychiatrists, together. CCPS was opportune and timely to the professional stage of the soon-to-graduate trainees in our series, who were transitioning

“…from working as skilled practitioners to teaching about skilled practice. Their experience and expertise must be rendered in forms that are accessible and meaningful to novices. What they used to do without thinking they must now do with thought to how it is understood by those watching and/or participating. As they work, they must translate that work into steps and language that help learners understand not only the skill of doing but also the inner workings of “what is going on here”” (32).

Though not explicitly construed as a seminar on leadership skills, CCPS did link to the psychology of leadership and to “well-held vulnerability” (48) as one of its integral components. Such an entreaty toward candid and shared vulnerability as a source of strength, rather than weakness, is not new. In his classic sociological text *Becoming Psychiatrists: The Professional Transformation of Self* , Donald Light had already noted how

“…more accurate than active-passive is the distinction between inner- and outer-directed; for the issue is not energy but susceptibility. All socialization requires active, emotional involvement; even when modeled, one is engaged. The passive person would be indifferent, apathetic” (49).

Even as we would be hard pressed to find an indifferent or apathetic participant to our CCPS series, and by that metric alone consider it to have been educationally effective, we go on to note the model's shortcomings.

### Limitations and Future Opportunities

CCPS is a logistics-intensive undertaking, as it requires, at a minimum, the availability of: (1) professional actors with experience in medical simulation; (2) funding, with costs that are not prohibitive yet important to take into consideration (we compensated actors at the institutional rate, with a minimum engagement of 4 h per session); (3) space, to which we had ready access, but which may not be a given at other sites; and (4) release time from clinical responsibilities for all trainees and faculty involved.

One way to approach the cost and logistic complexity of CCPS is through the creation of enduring materials such as videotaped interactions that can then be used as stimuli for discussion or as teaching materials. We have started to explore this approach by turning one of our CCPS sessions (VI, “sexual health discussions”) into an educational module we were able to deploy across a national network of CAP training programs (10). In this way, we have been able to consolidate and share training materials and standardized best practices across a larger number of sites and participants. Although this asynchronous (videotaped) approach does not address the same educational goals, we see it as a fruitful complement and natural byproduct of the synchronous CCPS model. The pandemic and the ensuing move to videoconferencing certainly contributed to this embrace of new technologies in education.

Our goal in this report was to explore the feasibility of applying the CCPS approach to psychiatry. This resulted in a 9R model of reflective practice that could in turn provide a useful rubric to support the academic and professional development of individual trainees and their personal milestones. Such a personalized approach was not our aim in this report but could be a fruitful next area of applied inquiry, both for shorter- (in-training) or longer-term (in-practice) educational outcomes.

We recognize that six of our seven simulations involved only one actor. Since clinical situations often involve several interacting individuals, we plan to explore these added layers of complexity in future adaptations of the model. None of our seven SPs was underage, a notable limitation when considering that our primary focus is on child and adolescent psychiatry. Even as children can be played by young adult actors, we are exploring ways of incorporating child actors into future scenarios (50). We are also interested in exploring the replication of a same case with an entirely new cast of participants, and to do so in a way that honors the “freshness” of each unique scenario.

Paradoxically, an approach that fosters spontaneity and rapid adaptation cannot be planned in as spontaneous and rapid a fashion. Indeed, advanced planning is inherently necessary to its process in order to recruit actors, to write scripts, and to get the activity ready for its learners. Despite these taxing requirements, we consider the potential to adapt CCPS to the specific local, linguistic, and cultural needs of its participants as one of its greatest potentials. For example, CCPS could be conducted in another language and, based on scripts developed on site, reflect the unique realities and idiosyncrasies of a given community and cultural context, applications of the approach that we intend to explore in the future.

## Conclusion

In summary, co-constructive patient simulation is an experiential approach that fosters autonomous, meaningful, and individually tailored learning opportunities. CCPS and the 9R model of reflective practice can be effectively applied to psychiatry and have the potential to contribute uniquely to the educational needs of its trainees and practitioners. By preparing them to “fail better,” CCPS seeks to humanize its participants in a generative way that not only celebrates and reaffirms their successes, but that fully embraces their imperfections and their unique feelings and fallibilities.

## Data Availability Statement

The raw data supporting the conclusions of this article will be made available by the authors, without undue reservation.

## Ethics Statement

This study involved human participants and was reviewed and approved by the Yale Human Investigations Committee (Protocol #2000026241). Written informed consent for participation was not required for this study in accordance with the national legislation and the institutional requirements.

## Author Contributions

AM and MC-F developed the CCPS model, they collaborated with IW, DA, and AA in scriptwriting and session preparation. AM, IW, DA, and MC-F designed the study and participated in its sessions. AM and IW took the lead in analyzing the data and drafted the first version of the manuscript. AM is responsible for the integrity of the data and analyses. All authors reviewed and contributed to working drafts and approved the final submitted version.

## Conflict of Interest

The authors declare that the research was conducted in the absence of any commercial or financial relationships that could be construed as a potential conflict of interest.
