# Supplementary material for: From Learning Psychiatry to Becoming Psychiatrists: A Qualitative Study of Co-constructive Patient Simulation
Source: Front Psychiatry. 2021 Jan 8;11:616239. doi: 10.3389/fpsyt.2020.616239 (PMC7820173; doi:10.3389/fpsyt.2020.616239)
Supplement: Supplementary file 1 [file Data_Sheet_1.PDF]

Confidential Training Document – Not for Distribution

Toriana 'Tory' Rae Jackson

Supplementary material

Case Author

Ayodola Adenike-Abike Adigun, MD MS

January 2020

Toriana 'Tory' Rae Jackson

General Objectives:

1. To explore how implicit and explicit biases can affect collaboration.
2. To provide an experiential opportunity to gain insight into effective communication and feedback in a supervisoral/ Doctor to Staff scenario.
3. To process how countertransference can positively and negatively impact interpersonal and interpersonal dynamics
4. To explore the role of a lead doctor when there is conflict with a staff member with a youth patient.

Case Summary

Scenario takes place in the nurse station at the back of a long term inpatient psychiatric hospital unit for adolescents.

Toriana 'Tory' Rae Jackson is a 33 year old single mother of a 3 year old boy, Benjamin who has special needs. Tory has been back to work for only a few months now as a youth coach on the unit. She is returning from a 3 month leave after being kicked in the back by a male patient during an aggressive restraint. She is reluctant to be back at work, but she does miss it- being at home and taking care of Benjamin was rough while dealing with her back pain. Benjamin's father is unemployed and has a history of incarceration. He does not provide financial support to Tory, nor does he visit his son. She is worried that not only does she have to learn the new, ever changing hospital policies, but also that the youth in the unit may not work well with her because she is 'new.' She has been a youth coach at the hospital for 2 years, but had difficulties feeling confident with her work because- ' there's always an issue.' Female staff members seem to collide with Tory, consequently, she speaks to mostly male staff members- some who she has interacted with intimately. Tory enjoys the unit gossip and sometimes partakes in talking to the patients about her personal life.

On a Sunday evening, Tory comes into the unit after being kicked in the abdomen by a male patient with Mild Intellectual Disabilities and a past psych history of DMDD, complex trauma- he is also currently exploring his gender identity. Tory was alone when this happened and had to call for back up which helped to redirect the patient. She asked for coverage so she could take time to write an incident report and speak to her supervisor. Her supervisor did not pick up. The patient who aggressed Tory is back on the unit and met with staff to process the altercation. He reported that Tory was 'being too personal' and that ' she asked [him] what his astrological sign was- ' she was in [his] business.' He admitted that he got frustrated during art therapy and ran out of the room. When Tory approached him he told her to ' get the f*ck' away from [him]. ' She cornered him while calling for back up, and when she started walking towards him, the patient flailed his hands at her. When she tried to stop him from flailing, he kicked her, right before back up arrived. Tory is upset that she got 'attacked

again.' She is helplessly looking for someone to speak to since her supervisor is not around. She also just heard from the lead milieu staff member that the patient got minimal consequences decided by the on call moonlighting psychiatrist.

Relevant Background

Toriana 'Tory' Rae Jackson is a 33 year old single mother. She was born the eldest of two to a single mom in Brooklyn, New York. Her family moved to New Haven when she was four to live with her maternal aunt. Tory's maternal uncle seemed to be especially fond of her - he would compliment her about her ' adult body', also telling her that her she had beautiful hair- ' the type that those girls buy at that hair salon for a fortune.' She was uncomfortable around him, but her mom always reassured her that , he was just being 'nice'. Tory was close to her mom when she was younger- she would always look forward to the night time when her mom would braid her hair. Tory's mom would tell her stories about her childhood, talk about her dreams that she could never fulfill, while brushing her her softly and attentively. Tory's mom would always tell her, ' Thank Dad for your good hair, mama's hair ain't sh*t.' Tory's mom smoked in the house, and survived off of food stamps and State assistance. Tory did not know her biological father- her mother always said that her brother looked like him, that was the only image she has of him, and she never pursued to find out more about him. Her relationship with her brother was initially good, but took a turn for the worse when he became a teenager and started to partake in gang activities. Tory tried to be a good sister to him, but he would always say, 'Mind your business, you're not my mom- she only cares about you anyways'. He eventually was convicted for attempted murder and tried as an adult, now serving 40 years in prison. Tory's mom smoking increased after her brother was sent to prison- she eventually died from Squamous Cell Lung Cancer.

Tory was able to graduate high school and receive an online degree- she wanted a better life. She acquired a Associate's Degree in Medical Assisting from Phoenix University online , and pursued various careers , finding her job as an inpatient youth coach at a long term psychiatric hospital for adolescents to be the most rewarding. Tory looked for a State job, mainly to be supported by the benefits, but also because she heard that ' they rarely fire.' She was initially fired from her first job as a preschool assistant for incessantly falling asleep on the job. Her second job as a direct support staff member at a State subsidized residential for adults with developmental disabilities was curtailed after 2 years when a resident complained of verbal and physical abuse and was noted to have bruises around her upper arm. Tory was adamant about the allegations, and attempted to get a pro bono lawyer from the Statewide Legal Services of Connecticut. After being told that she was found on camera aggressing the patient, she declined her pursuit.

For 8 months, Tory was out of work, and partook in focus groups, and craigslist gigs ranging from house cleaning to brand promoting in order to financially manage. She found herself worrying about money week to week, having to borrow money from friends for work. Tory found herself contacting ex's during this time as well, not only for money , but also for intimate comfort as she was lonely. The sex was disconnected and

became a form of self medication, and escape from her harsh reality. Tory did not stop applying for jobs, but seemed to never get a call back. Tory was applied for food stamps, and was able to find some financial relief. Amid her jobless course, Tory would passively use Tinder to strike conversations with males and females. She connected with a male, Christopher (Chris) ' Butterfly' Walters, who not only was physically attractive, but appeared to be in better phase of life than Tory. She enjoyed conversation with Chris, finding herself texting him throughout the day, and as a night cap. He told Tory that he was a Pharmaceutical Representative - she never asked him too much into his job, but when she did, he would switch the subject abruptly- Tory never thought too much of it. Chris would always tell her how pretty she was, he always had the right thing to say. Every insecurity that Tory had about herself, Chris repudiated with soft complements and future oriented, hypnotizing promises. They eventually exchanged numbers and arranged to meet at the local diner on a date. To Tory's amazement, Chris was more than she imagined- He stood 6'5", arms perfectly delineated, conspicuously amplified with his tight long sleeve muscle shirt, groomed to a T. And his eye contact- Tory ' never found a man who looked at [her] like that.' Chris had an interesting butterfly tattoo on his eye, he told Tory he got it ' during a time where he was not free, but wanted to feel so." When Chris first met Tory, he told her that she was ' just right.' He told her that she had ' good hair', the type that could play whatever role it was given. Tory and Chris went on a few other dates, and he would always communicate about her physical qualities, sometimes making her feel uncomfortable with this new attention, but always eventually comforted with, " You deserve it." Things started to move fast with the two, and Chris was able to convince Tory to explore exotic dancing on the side, for financial stability. He offered to get Tory a gig, and just asked for a percentage of her weekly accruements, which he titled a ' finder's fee.' Tory was reticent to the idea at first, she never danced, never was a performer, and the idea of doing both with minimal to no clothing was something she thought she could never do. Chris did follow through and found her a position at one of the ' better gentlemen's club' in the city- he helped her conjure some courage to try her first night. Tory was nervous, but seeing Chris there, helped bring her to ease. After her first night, Tory made $2,500- she never made money like that before! She became addicted to dancing. Chris never left her side, but was also prompt to ask for his cut at the end of the week.

After making adequate money at her dancing job, Tory was able to regain some confidence to reapply to jobs- she didn't want to do this all her life. Chris did not like the ideas of this, and tried to convince her otherwise. He started to show her a different side- now not-so sweet, curt, and even the way he looked at her changed. Chris and Tory were intimate and would not use protection during sex- Chris insisted it was ok , and that he was not with other people. Tory started gaining weight, and was not bringing in as much money as she used to. Chris also didn't seem to be as physically attracted to her as he used to. Tory was surprised to find out that she was indeed pregnant, after realizing that her period did not come for 2 months. As soon as she found out, she called Chris- but he was no where to be found. After weeks' worth of missed calls and unreturned texts, Tory started to wonder who Chris really was. She searched him

online, and to her dismay, found out that Chris was an ex- convict. He was in and out of jail for drug possession and money laundering. Tory was heartbroken. Not only was money low again, but she now found her self even more lonely, but this time with a child on the way. Tory desperately reached out to extended family for help- no-one ever responded. She found herself backed up in the corner, contemplating ending her life, but what seemed to keep her going was hearing Chris' voice telling her ' You can do anything with that good hair.' She never hated herself more, but decided to keep her life to try to give her child a better chance at living.

Tory's friend mentioned to her a new gig as a youth coach at a long term psychiatric hospital , she encouraged Tory to apply- 'its easy, you are protected by the State, get great benefits, and you known how the state is, they never fire!' Tory applied for the job and to her disbelief she got it. Tory did not find the job too hard as she worked with kids before, she also had some experience with working in the mental health field. Although working while pregnant was difficult, she was relieved to have something to look forward to- a job which did indeed guarantee her and her child a better future.

Tory took maternity leave and gave birth to her son, Benjamin. Benjamin was born with Down Syndrome and Tory experienced much difficulty during his infancy. She was able to contact Chris to inform him about his son, but he remained distant. One night, Chris randomly showed up to her house demanding to see his son, and Tory found herself unreasonably happy to see him- she was in love with him after all. Chris looked different, skinnier. Tory and Chris had sex that night, but in the morning Chris was nowhere to be found. Tory reveled over the night, and seemed to snap out of Chris's absence quicker than she thought- finding herself in a better place emotionally. She was able to find in home care for her son provided by the State, as she was eager to go back to work.

When Tory returned back, she felt ' different' and not as close to the staff as she though she was, especially the females. She found herself more comfortable with the males staff- as "the females on the unit were messy and jealous of me, maybe because I am prettier than them- I'm sure they hate me because of my good hair ." After pregnancy her hair grew even more, it has healthier and longer than the mane of a prized stallion- it truly was the only thing she valued. Tory would confide with the male staff about her day to day, and even pursued external intimate relationships with some of them, adding 'adventure to her boring, rote life. With the other men, she demanded the use of protection as she didn't want another child. Her supervisor at times would tell her that she was talking too much to the male staff , and not paying enough attention to the patients. As a coach, Tory would talk to the patients about her personal life, sometimes too personal- also asking the patients intrusive questions, blurring the lines professionalism. Often, Tory would partake in gossip with the patients about happenings on the unit- ' she just wanted the kids to like [her]'. On one occurrence, Tory was called to help with a restraint on the unit for a dysregulated youth. When the youth saw that she was coming to help, he spat in her face and kicked her in the back, accusing her of

previously talking about his mother. Tory's consequently injured on the job, and she was sent on medical leave with pay for 3 months.

During the 3 months Tory's in home assistance hours were reduced, and taking care of Benjamin on her own was hard, encumbered by her back pain. She found little relief with her high strength Tylenol, however encountering mitigation of pain with CBD pens and oils. Tory started to lose weight, and feeling flu symptoms, but she attributed it to her stress level, until a friend convinced her to go to the doctor for a check up. Disgruntedly, Tory went and received the worse news of her life- she was HIV positive. She was destroyed- not knowing who gave it to her or when- however able to eventually narrow it down to Chris- he was the only one who she has unprotected sex with after all. Tory sent Chris a text asking if it was him, and to her surprise he replied saying- " Im sorry." Tory was started on antiretroviral therapy, but hated everything about taking medication. She became depressed, and eventually was noncompliant with the medication.

Tory's only relief was at work- after medical leave she returned back to the youth coaching job. She informed the male staff who she previously had sex with of her new diagnosis urging them to get a test, but also making them promise to tell anyone. They were all negative. None ever told. Tory found herself robotically going from work to home- fatigued. With the in home services back up now that she has returned, she started to distance from Benjamin, locking her self in the room, guilty and terrified of what the future will hold. Tory wore a mask to work, despite a few comments on her losing weight, no one was able to tell that she was indeed suffering. She found herself crying in the bathroom on breaks, walking back out, however, with new eyeliner, and with her ' good hair, ' rebrushed. Tory hated who she saw in the mirror.

A few months after returning, she decided to do a double on the weekend - Christmas was near after all, and she wanted to at least be able to buy Benjamin the gifs he deserved. On her shift, Tory was kicked in the abdomen by a male patient, named ' Chris' with Mild Intellectual Disabilities and a past psych history of DMDD, and complex trauma- this patient was also currently exploring his gender identity. Tory followed the patient for safety coverage when he ran into the hallway after being frustrated during art therapy. She was alone when this happened and had to call for back up . She cornered him while calling for back up, and when she started walking towards him, the patient flailed his hands at her. When she tried to stop him from flailing, he kicked her in the abdomen, right before back up arrived. Tory was upset that she got 'attacked again.' She incessantly repeated , " Chris did it , Chris did it, I am tired of this happening to me." She tried to page her supervisor, but she as nowhere to be found. She was frazzled writing an incident report, but was able to complete it through angry tears. Tory helplessly looked for someone to speak to since her supervisor was not around- she needed someone to listen to her.

"No one is ever there when I need them." She also just heard from the lead milieu staff member that the patient got minimal consequences- hallway restriction for the night- decided by the on call moonlighting psychiatrist. When she comes in the unit,

she is frazzled. She attempts to confront the moonlighting doctor about his/her decision to give the patient who attacked her a minimal consequence. When asked about the situation, she mentions that the patient kicked her in the abdomen and stated that she did not approach him, although he stated otherwise. She makes it a point to mention that he scratched her on the face, noticeably insistent that there was no blood exchange. She is changing positions a lot during the conversation, first standing then finding her self seated , but with obvious restlessness. She is insistent that she has worked with kids before, especially kids with Autism and learning disabilities so she is convinced she did not do anything wrong. She accuses the staff of not liking her, and of the patients also talking about her, suggesting that they're just jealous of her, because of her 'good hair'. She can not seem to let go of the idea that the patient's name is Chris too. When asked how she could be helped, Tory states that no-one can help her- she then goes on a tirade sharing that she has had a difficult past and that people think she has a chip on her shoulder, but she feels that others just don't care to understand her. She expresses presently feeling stressed, losing weight, forgetting things with provided examples at home and work, and just feeling sick overall- with a bad, unremitting flu. She is coughing intermittently. When asked about her HIV status, she tears up, but then admits that she has not been compliant with her meds, and she really does not know what to do. Once told of the need of the moonlighting doctor to speak to write an incident report of the events that transpired during the night, she becomes angry, stating again once again she can not trust anyone. She is however, eventually able to be consoled, and is encouraged to speak to her supervisor/medical director about her current condition.

Toriana 'Tory' Rae Jackson

Door Note:

Scenario takes place in the nurse station at the back of a long term inpatient psychiatric hospital unit for adolescents.

Toriana 'Tory' Rae Jackson is a 33 year old single mother of a 3 year old boy, Benjamin who has special needs. Tory has been back to work for only a few months now as a youth coach on the unit. She is returning from a 3 month leave after being kicked in the back by a male patient during an aggressive restraint. On a Sunday evening, Tory comes into the current unit you are moonlighting at after being kicked in the abdomen by a male patient with Mild Intellectual Disabilities and a past psych history of DMDD, and complex trauma- he is also currently exploring his gender identity. Per staff report, Tory was alone when this happened and had to call for back up which helped to redirect the patient. The patient who aggressed Tory is back on the unit and met with you to process the altercation. He reported that Tory was 'being too personal' and that ' she asked [him] what his astrological sign was- ' she was in [his] business.' He admitted that he got frustrated during art therapy and ran out of the room. He stated that when Tory approached him, he told her to ' get the f*ck' away from [him]. ' She cornered him while calling for back up, and when she started walking towards him, the patient flailed his hands at her. When she tried to stop him from flailing, he stated that he motioned to kick her before back up arrived, but never hit her. Tory is upset that she got 'attacked again.' She is helplessly looking for someone to speak to since her supervisor is not around. She also just heard from the lead milieu member that the patient got minimal consequences- hallway restriction for the rest of the night - decided by you.

Intended direction of SP interaction

1. **Learners should be able to get the relevant history of Tory's report of the incident- exploring her perspective and contributing history to her current presentation with empathy and active listening.** *(SP: be open with details of altercation, perseverant about topics of a tough past, 'good hair', and perceptive about how others 'view' her. Be guarded of current health symptoms)*

   While maintinaing a non-judgmental stance, the learner should be able to ask salient, situation-related, non-blurring boundary questions to understand and emphatize with Tory's experience, and to be able to make a clear decision of the direction of action with Tory, the youth, and the hospital.

2. **Learners should do a risk assessment with emphasis on Tory's wellbeing. Tory reveals that she is HIV positive.** *(SP: the second interviewer should arrive at this information, be more open- give more hints about symptoms)*

The learner, assuming a supervisorial role, should be able to navigate his/her understanding of hospital systems/dynamics including – patient care, staff support, and incident reporting in order to professionally guide his/her questioning to maintain a non discminatory, legal interaction with the staff member. The clinician can not directly ask the staff member what her HIV status is, as in accordance to the American Disabilty Act. After Tory voluntarily informs the learner about her HIV+ status, the learner can ask about potential safety risks during incident with the youth (i.e., blood exchange).

3. **Learner should be able to provide feedback to Tory about her current situation, also emphatically acknowledge her concerns. Tory should be advised to speak to her medical director/supervisor about current health condition, also be informed that an incident report/note will be written up about her interaction with the patient. Tory should be reassured that this conversation is confidential** *(SP: Tory's current HIV status should be revealed, with worry about her and son's future also communicated, Tory is eventually able to be consoled and reassured after persistent empathy)*

The learner can advise Tory to tell her workplace, but should be told that she does not have to do so after safety has been ascertained. She should also be educated that if she does not inform the hospital of her condition, but there is objective evidence that she can not do her job or if she poses a safety risk, her employer can ask medical questions.

Her HIV+ status can not be mentioned in the incident report written by the learner. In accordance to the American Disability Act, when Tory asks about consequences of telling the hospital of her HIV+ status, she should be told that she can not be fired because of her status; also that she can ask for reasonable accomodations.
